# Stereotactic radiotherapy is a useful treatment option for patients with medullary thyroid cancer

**DOI:** 10.1186/s12902-021-00832-4

**Published:** 2021-08-09

**Authors:** Aleksandra Kukulska, Jolanta Krajewska, Zofia Kołosza, Aleksandra Grządziel, Mateusz Gajek, Ewa Paliczka-Cieślik, Dorota Syguła, Kornelia Ficek, Aneta Kluczewska-Gałka, Barbara Jarząb

**Affiliations:** 1 Nuclear Medicine and Endocrine Oncology Department, Maria Sklodowska-Curie National Research Institute of Oncology Gliwice Branch, Wybrzeze AK 15, 44-101 Gliwice, Poland; 2 Radiotherapy Department, Maria Sklodowska-Curie National Research Institute of Oncology Gliwice Branch, Wybrzeze AK 15, 44-101 Gliwice, Poland; 3 Department of Biostatistics and Bioinformatics, Maria Sklodowska-Curie National Research Institute of Oncology Gliwice Branch, Gliwice, Poland; 4 Radiotherapy Planning Department, Maria Sklodowska-Curie National Research Institute of Oncology Gliwice Branch, Gliwice, Poland

**Keywords:** Medullary thyroid carcinoma, Radiosurgery, Stereotactic radiotherapy, Radiation treatment, Palliative radiotherapy

## Abstract

**Abstract:**

The role of radiotherapy in advanced medullary thyroid carcinoma (MTC) is confined to patients in whom surgical treatment or the administration of tyrosine kinase inhibitors are not possible or contraindicated. High fractionated radiation doses during radiosurgery or fractionated stereotactic radiotherapy are applied to reduce cancer-related symptoms and stabilize irradiated lesions. This study aimed to retrospectively evaluate the therapeutic effect of stereotactic radiotherapy in MTC patients.

**Material and methods:**

The study group involved 11 MTC patients, treated due to 16 cancer lesions, mainly bone metastases (10 lesions), lymph node (2 lesions) metastases, or liver metastases (2 lesions), one primary thyroid tumor, and one MTC recurrence in the thyroid bed. The fractionated and total radiation doses ranged between 5 and 12 Gy and 8–44 Gy, respectively. Six lesions were treated with a single radiation fraction, three lesions with 2 fractions, another 6 lesions with 3 fractions, whereas the remaining one metastatic lesion with 9 fractions of stereotactic radiosurgery.

**Results:**

The beneficial effect of stereotactic radiosurgery was obtained in all treated lesions. None of treated lesions progressed in the further disease course. Fourteen lesions were stable (87.5 %), including eight lesions showing progression before radiosurgery (good response). Disease control was obtained in all soft-tissue metastases. Regarding bone metastases, partial regression was achieved in 20 % lesions, whereas in 30 % lesions progressive before radiotherapy, the treatment led to disease stabilization.

**Conclusions:**

Our data pointed to the effectiveness of high-dose fractionated radiotherapy in MTC. However, an observation of a larger group of patients is required to confirm it.

## Background

Medullary thyroid carcinoma (MTC), a rare malignant neoplasm arising from C cells, represents less than 5 % of all thyroid cancers. It may occur as a sporadic disease or part of a hereditary multiple endocrine neoplasia (MEN) syndromes [1–5]. Surgery based on total thyroidectomy and neck lymph node dissection is the main and most effective treatment option. Other standard oncological treatment modalities, including chemo and radiotherapy, demonstrate a limited efficacy in MTC [6].

The role of radiotherapy in MTC has been confined to patients in whom surgical treatment or the administration of tyrosine kinase inhibitors are not possible or contraindicated. Published data regarding the effectiveness of radiotherapy are equivocal [7–11]. It is routinely applied as palliative therapy. The current ATA (American Thyroid Association) guidelines recommend adjuvant radiotherapy in patients with a high risk of local recurrence [6]. Our previous paper showed that the effect of postoperative adjuvant radiotherapy with fractionated doses of 2 Gy did not significantly impact the risk of local relapse [12]. Higher fractionated radiation doses are usually applied in patients with metastatic disease to reduce cancer-related symptoms and stabilize irradiated lesions [13]. The administered fractionated dose is limited by the necessity to protect critical organs localized near irradiated lesions. We tend to use the highest radiation doses in the shortest time that are not related to the risk of radiation complications. Such an approach improves the patient’s comfort as well as treatment effect.

Radiosurgery (single radiation dose) or fractionated stereotactic radiotherapy (few radiation doses), allowing for a precise concentration of a high radiation dose, fulfill the above-mentioned conditions [14]. Thanks to the appropriate selection of a large number of beams and frequent monitoring of the patient’s position during a radiotherapy session, precise treatment is possible. Many beams from different directions are concentrated in the middle of the treated volume, providing a very high radiation dose, higher than the doses used in conventional treatment. In comparison, the use of frequent imaging of the patient with the kV radiation provides monitoring of selected anatomical structures and assessing their position with respect to the planned position. Maintaining the unchanged position of anatomical structures, including the target, relative to radiation beams, allows for delivering a high fractional dose and limitation of treatment to one or several sessions.

The systems consist of a linear accelerator mounted on a robotic arm, repositionable couch, x-ray cameras, and imaging panels. For moving targets, the synchrony camera is used.

From the classic C-arm accelerators, the CK system differs in the way the accelerator is moved. Many treatment beams can enter the patient’s body from different directions by placing an accelerator on the robot. In the conventional accelerator, the gantry can rotate in one plane only. Moreover, in the CK system, patient imaging (2D kV) is performed during the therapy session, between the beams. It is an automated process performed even several dozen times per fraction. Thus, the precision of treatment is ensured by frequent imaging of the patients and their repositioning. In moving tumors, the CK system provides synchronization of the accelerator movement with the patient’s breathing. It is different from stereotactic treatment on classic accelerators, where mostly 3D before irradiation is performed.

Two main features of the CK system, i.e., the use of several hundred beams and frequent x-ray patient imaging during therapy, cause the session to last longer than on a conventional accelerator.

Our study aimed to evaluate the therapeutic effect of stereotactic radiotherapy in MTC patients.

## Methods

The study group involved 11 MTC patients, treated due to 16 cancer lesions, subjected to a retrospective analysis. Eleven lesions were subjected to CK, whereas the remaining five lesions were irradiated on a conventional linear accelerator, including one patient with respiratory gating. The fractionated and total radiation doses ranged between 5 and 12 Gy and 8–44 Gy, respectively. Six lesions were treated with a single radiation fraction, three lesions with 2 fractions, another 6 lesions with 3 fractions, whereas the remaining one metastatic lesion with 9 fractions of stereotactic radiosurgery. We treated mainly bone metastases (10 lesions), lymph node metastases (2 lesions), liver metastases (2 lesions), one primary thyroid tumor, and one MTC recurrence in the thyroid bed (Table 1 – in a separate file). Apart from the symptoms related to the tumor itself, the indication for the treatment was disease progression confirmed by imaging studies.

**Table 1:** Characteristics of the study group

Patient	sex	age	pT	pN	M	target	Treatment method	Treatment date	Fractionated Dose [Gy]	Total dose [Gy]	Follow-up [years]
1.	m	56	2	1	LN, bones, lungs	Th1 Th10	CK CK	2012, 2018	10 6	10 18	7 1
2.	f	44	x	1	bones	Th7 L4	CK CK	2016 2016	10 10	30 30	3 3
3.	f	65	x	1	bones	Th8	ST		10	10	8
4.	f	75	x	1	LN, bones liver	Liver	ST-G	2019	12	12	1
5.	f	39	2	0	bones, liver	L4 Th2	CK CK	2012 2011	8 8	16 16	8 9
6.	f	60	3	1	bones, liver	Skull Liver	CK CK	2012 2012	10 8	10 8	1 1
7.	f	65	x	1	LN, liver	LN	CK	2018	8	24	1
8.	m	44	4	1	bones	Th6 L1	CK CK	2013 2013	7 10	21 20	1 1
9.	f	62	4	1	Lungs, local recurrence	Recurrence	St	2006	8	8	2
10.	m	24	1	0	0	Primary thyroid tumor	St	2017	12	36	1
11.	m	50	3	1	Retropharyngeal LN	LN	CK	2016	5	45	3

*LN* lymph nodes, *CK* cyber knife, *St* Stereotactic radiotherapy

Treatment response was evaluated by imaging studies. We evaluated the dynamics of bone lesions using CT, whereas the dynamics of local recurrence, lymph node and liver metastases were evaluated according to RECIST criteria based on CT and MR, respectively. The result of the most recent imaging assessment following stereotactic radiotherapy, performed before another therapeutic procedure, was considered an outcome. Stabilization of a lesion progressing before radiotherapy or tumor size reduction was considered a good response.

Follow-up ranged between 1 and 9 years, mean was 3 years, median 1.5 years.

The study was approved, and the need of informed consent was exempted by the Bioethical Committee at M. Sklodowska-Curie National Research Institute of Oncology, Gliwice Branch (KB/430 − 72/20) as the informed consent was not required by the bioethical committee.

Stereotactic plans were calculated in the BrainScan v. 5.31 (BrainLAB GmbH, Heimstetten, Germany) treatment planning system. Then successively, the latest versions of the software were used: iPlan v.3.0 since 2007 and iPlan v. 4.5 since 2011.

Treatment plans were implemented at Clinac 2300 Varian Medical Systems (Palo Alto, CA, USA) with the attached to the gantry. The m3 µMLC was developed jointly by BrainLAB GmbH and Varian Medical Systems. The m3 had 14 pairs of 3 mm leave in the center of the field, 6 pairs of 4.5 mm leave in the middle, and six pairs of 5.5 mm leave at the periphery for a maximum field size of 9,8 × 9,8 cm^2^. The dimension of the lives is defined in the isocenter.

Since the CK VSI system (Accuray, Sunnyvale, CA, USA) installation in 2010, stereotactic plans can also be delivered in a robotic manner. The CK system is equipped with two types of circular collimators: fixed and Iris variable aperture, both with diameters from 5mm up to 60mm. Our Institute has also been using the modern CK M6 system, additionally equipped with a multi-leaf collimator, since 2018.

Dose distribution calculations are performed in two treatment planning systems: MultiPlan v. 4.6 and Precision v. 2.0 dedicated to VSI and M6 units, respectively.

Statistical analysis, carried out using Dell Inc. (2016) Dell Statistica version 13 and Stata/MP 13.1 for Windows, was based on the following tests: the exact Fisher’s test and Mann-Whitney test.

## Results

Stabilization after stereotactic radiosurgery was observed in the further disease course in all treated lesions. Fourteen lesions were stable (87.5 %), including eight lesions showing progression before radiosurgery (good response). While the volume of two other lesions significantly reduced. Good treatment response was obtained in 62.5 % of lesions (10 out of 16). Treatment outcomes are summarized in Table 2.

**Table 2:** Summary of radiotherapy outcomes

Patient	Target	Treatment date	Progression prior to radiotherapy	Outcome	Fractionated dose	Total dose
1.	Th1 Th10	2012, 2018	No No data	SD No data	10 Gy 6	10 18
2.	Th7 L4	2016 2016	No No data	SD	10 10	30 30
3.	Th8	2008	No	SD	10	10
4.	Liver	2019	YES	SD	12	12
5.	L4 Th2	2012 2011	No No data	PR PR	8 8	16 16
6.	Skull Liver	2012 2012	YES YES	SD No data	10 8	10 8
7.	LN	2018	YES	SD	8	24
8.	Th6 L1	2013 2013	YES YES	SD SD	7 10	21 20
9.	Recurrence	2006	No	SD	8	8
10.	Primary thyroid tumor	2017	YES	SD	12	36
11.	LN	2016	YES	SD	5	45

*SD* stable disease, *PR* partial response

Regarding bone metastases, partial regression was achieved in 2/10 (20 %) lesions, whereas in 3/10 (30 %) lesions, progressive before radiotherapy, the treatment led to disease stabilization.

Disease control was obtained in all soft-tissue metastases. 83 % of them (5 lesions) demonstrated progression before radiosurgery.

Fractionated (*p* = 0.77) and cumulative (*p* = 0.586) radiation doses did not significantly differ between the group with a good response (PR or SD in progressive lesions) and the remaining patients (Table 3).

**Table 3:** Radiotherapy doses [Gy] with reference the treatment outcome

	Fractionated dose [Gy]			Total dose [Gy]		
	Mean + SD	median	range	Mean + SD	median	range
Good treatment response (regression or stabilization of lesion progressed before radiotherapy	8.8 + 2.2	8	5-12	20.8 + 11.7	18	8-45
Remaining patients (disease stabilization)	9 + 1.7	10	6-10	17.7 + 10.02	14	8-30
The whole group	8.9 + 2.0	9	5-12	19.8 + 10.9	17	8-45

There were no significant differences in outcomes regarding the localization of metastases (*p* = 0.44; Table 4).

**Table 4:** Localization of irradiated lesions

Localization of irradiated lesions	Number of lesions showing good treatment response	The whole group
Bones	5 (50 %)	10
Lymph nodes	2 (100 %)	2
Liver	2 (100 %)	2
Recurrence in thyroid bed	0 (0 %)	1
Primary thyroid tumor	1 (100 %)	1

In one patient (patient nr 10), stereotactic radiosurgery was the only therapeutic method of the primary, low advanced MTC tumor, which was not amendable for surgery due to important comorbidities. Stereotactic radiotherapy resulted in the stabilization of a previously progressive tumor and a decrease in serum calcitonin concentration.

There were no treatment-related radio-necrosis and any other severe complications observed during the further follow-up.

## Discussion

Our results demonstrate a beneficial effect of high-dose fractionated radiotherapy in MTC. However, a low number of analyzed patients did not allow us to draw definite conclusions. The rare MTC occurrence, especially in its advanced form, makes it impossible to collect a larger study group. However, one should notice, that our patients constitute one of the larger groups analyzed. The published data usually concerned with single MTC cases. Bernstein et al., who reported the outcomes of phase I/II trial concerning stereotactic radiotherapy in treating thyroid cancer vertebral metastases, involved only 4 MTC patients [15]. Boyce-Fappiano et al. analyzed a group of 67 thyroid cancer patients, 10 of which were medullary thyroid carcinoma cases. Stereotactic radiosurgery was used for metastatic spine tumors [16]. Other papers described single MTC patients with brain metastases [17–20]. Bernard et al. emphasized that the beneficial effect of radiosurgery was observed mainly in differentiated thyroid carcinoma [19]. The other authors reported good treatment outcomes considering the whole analyzed groups, with MTC patients comprising more than 20 % of patients.

The highest percentage in our group constituted patients with bone metastases. Two metastatic bone lesions decreased their volume after radiotherapy, whereas the remaining eight bone lesions were stable, including three metastases with progression before radiosurgery. Similar data were reported by Bernstein et al. [15], who analyzed 23 patients, among them 4 MTC cases. Moreover, Boyce-Fappiano et al. analyzed a large group of patients and observed a good response for spine stereotactic radiotherapy in ten metastatic thyroid cancer cases [16]. The ability to stabilize spinal metastases is crucial because progression could lead to spinal cord injury, significantly worsening patients’ quality of life. In addition to spinal metastases, tumors in the neck are the second group of lesions whose progression can cause significant damage to the patient’s health. Timely radiotherapy can prevent dysphagia and shortness of breath caused by tumor expansion in the neck.

There were six soft-tissue metastases in our group, in which high-dose stereotactic radiotherapy resulted in beneficial outcomes. Similar effects were observed by Kim et al., who applied stereotactic radiosurgery in 9 patients with lymph node metastases in the course of thyroid carcinoma, among them 2 MTC cases [17]. The effectiveness of stereotactic radiotherapy in treating soft tissue metastases was confirmed by Ishigaki et al., who treated 25 patients diagnosed with differentiated thyroid cancer due to local recurrence not amendable for surgery [21]. Radiotherapy led to disease stabilization in all treated patients.

Although our group is not large enough to undergo a complete statistical evaluation and draw clear conclusions, it provides information for the clinician indicating the possibility of treatment of single MTC foci that cannot be removed surgically. Nevertheless, one should stress, our study group consisted only of MTC patients. It allowed us to conclude on the usefulness of stereotactic radiotherapy in treating these patients with somewhat more certainty than in the case of mixed groups as MTC biology and treatment methods differ from other types of thyroid cancer. So, a pooled analysis of all thyroid cancer patients may result in imprecise conclusions. It would be valuable to be able to confirm our results on a larger group of patients.

We believe our study will be helpful in clinical decision-making regarding the management of patients with metastatic or locally advanced medullary thyroid cancer, supporting the clinician in the treatment of patients for whom radiotherapy may be the only feasible treatment modality. The short time, satisfactory efficacy, and the lack of severe treatment-related complications of stereotactic radiotherapy allow applying it in patients in a more severe general condition, not amendable for surgery or systemic treatment.

## Conclusions

To conclude, our data pointed to the effectiveness of high-dose fractionated radiotherapy in MTC. However, an observation of a larger group of patients is required to confirm it.

## Data Availability

The datasets used and analyzed during the current study are available from the corresponding author on reasonable request.

## Abbreviations

ATA: American Thyroid Association
CBCT: Cone-beam computed tomography
CK: Cyber-knife
CT: Computed tomography
MEN: Multiple endocrine neoplasia
MRI: Magnetic resonance
MTC: Medullary thyroid carcinoma
PR: Partial regression
SD: Disease stabilization
μ MLC: Micro multi-leaf collimator
